# Malnutrition in Sub – Saharan Africa: burden, causes and prospects

**DOI:** 10.11604/pamj.2013.15.120.2535

**Published:** 2013-08-06

**Authors:** Luchuo Engelbert Bain, Paschal Kum Awah, Ngia Geraldine, Njem Peter Kindong, Yelena Sigal, Nsah Bernard, Ajime Tom Tanjeko

**Affiliations:** 1 Braun School of Public Health and Community Medicine, Hebrew University of Jerusalem, Israel - Centre for Population Studies and Health Promotion, CPSHP, BP 7535, Yaoundé, Cameroon; 2 Faculty of Arts, Letters and Social Sciences, FALSS, University of Yaoundé I, Cameroon; 3 Faculty of Science, University of Dschang, Cameroon; 4 Awing District Hospital, Santa Health District, Bamenda, Cameroon; 5 Faculty of Health Sciences, University of Buea, Cameroon

**Keywords:** Malnutrition, Sub–Saharan Africa, corruption, multisectorial approach

## Abstract

Malnutrition is estimated to contribute to more than one third of all child deaths, although it is rarely listed as the direct cause. Contributing to more than half of deaths in children worldwide; child malnutrition was associated with 54% of deaths in children in developing countries in 2001. Poverty remains the major contributor to this ill. The vicious cycle of poverty, disease and illness aggravates this situation. Grooming undernourished children causes children to start life at mentally sub optimal levels. This becomes a serious developmental threat. Lack of education especially amongst women disadvantages children, especially as far as healthy practices like breastfeeding and child healthy foods are concerned. Adverse climatic conditions have also played significant roles like droughts, poor soils and deforestation. Sociocultural barriers are major hindrances in some communities, with female children usually being the most affected. Corruption and lack of government interest and investment are key players that must be addressed to solve this problem. A multisectorial approach is vital in tackling this problem. Improvement in government policy, fight against corruption, adopting a horizontal approach in implementing programmes at community level must be recognized. Genetically modified foods to increase food production and to survive adverse climatic conditions could be gateways in solving these problems. Socio cultural peculiarities of each community are an essential base line consideration for the implementation of any nutrition health promotion programs.

## Introduction

Malnutrition is estimated to contribute to more than one third of all child deaths, although it is rarely listed as the direct cause [1]. Malnutrition literally means “bad nutrition” and technically includes both over- and under- nutrition. The World Food Programme (WFP) defines malnutrition as “a state in which the physical function of an individual is impaired to the point where he or she can no longer maintain adequate bodily performance process such as growth, pregnancy, lactation, physical work and resisting and recovering from disease”[1]. Contributing to more than half of deaths in children worldwide; child malnutrition was associated with 54% of deaths in children in developing countries in 2001 [2, 3]. Protein-energy malnutrition (PEM), first described in the 1920s, is observed most frequently in developing countries but has been described with increasing frequency in hospitalized and chronically ill children in the United States [4].

Poor environmental conditions may increase insect and protozoan infections and also contribute to environmental deficiencies in micronutrients. Overpopulation, more commonly seen in developing countries, can reduce food adequacy, leading to inadequate food intake or intake of foods of poor nutritional quality and quantity. Conversely, the effects of malnutrition on individuals can create and maintain poverty, which can further hamper economic and social development [3]. This is explained with children starting life with low intellectual quotients and being impossible later to offer the best of their expected intellectual abilities.

Kwashiorkor and marasmus are two forms of Protein Energy Malnutrition (PEM) that have been described. The distinction between the two forms of PEM is based on the presence of edema (kwashiorkor) or absence of edema (marasmus). Marasmus involves inadequate intake of protein and calories, whereas a child with kwashiorkor has fair-to-normal calorie intake with inadequate protein intake. Although significant clinical differences between kwashiorkor and marasmus are noted, some studies suggest that marasmus represents an adaptation to starvation whereas kwashiorkor represents a dys-adaptation to starvation.

In addition to PEM, children may be affected by micronutrient deficiencies, which also have a detrimental effect on growth and development. The most common and clinically significant micronutrient deficiencies in children and childbearing women throughout the world include deficiencies of iron, iodine, zinc, and vitamin A and are estimated to affect as many as two billion people. Although fortification programs have helped diminish deficiencies of iodine and vitamin A in individuals in the United States, these deficiencies remain a significant cause of morbidity in developing countries, whereas deficiencies of vitamin C, B, and D have improved in recent years. Micronutrient deficiencies and protein and calorie deficiencies must be addressed for optimal growth and development to be attained in these individuals.

## Methods

We searched medical literature in biomedical databases PUBMED, OVID and Google scholar using the following key words: “Malnutrition”, “Burden”, “Determinants”, “Causes”, and “Sub - Saharan Africa”. The search was limited to articles published in and after 1993. The bibliographies of the articles on hand were used to find other references. We also searched through indexes of major journals that publish on malnutrition in Sub-Saharan africa. Of the 267 articles we found only 29 were included in the final review. These were articles that had data on determining factors and burden of malnutrition in Sub-Saharan Africa. A critical analysis that entailed systematic review of these articles in Sub Saharan-Africa, relevant to malnutrition information was done.

## Current status of knowledge

### Context and Rationale

The impact of malnutrition usually falls mainly on children under five years of age [1]. Conceptually speaking, malnutrition generally involves nutrition and obesity. Africa is going through a rapid sociodemographic transition, with an alarming increase in incidences of obesity, diabetes mellitus, cardiovascular diseases (stroke and myocardial infarction). Our main focus will be under nutrition. Despite the millennium development goals target to reduce hunger by half by 2015, major failures have been recorded mainly in Africa. Out of the 800 million people still suffering from hunger in the world, over 204 million come from Sub-Saharan Africa. The situation is currently getting worse in this region as it moved from 170.4 million hungry people in 1990 to 204 million in 2002 [5]. This increase has generally been attributed to poverty, illiteracy, ignorance, big family size, climate change, policy and corruption. Infectious diseases are the major cause of mortality and morbidity in developing countries. PEM is also associated with a number of co morbidities such as lower respiratory tract infections including tuberculosis, diarrhea diseases, malaria and anemia. These co-morbidities may prolong the duration of hospital stay and death among affected children [6, 7].

### Food security in Sub-Saharan Africa

Food security is said to exist if at all times, people have a physical and economic access to sufficient, safe and nutritious food that meet their dietary and food preferences, for an active and healthy life [8]. This ongoing condition has been caused by a number of factors including distribution obstacles, global climate change, a lack of successful local agriculture, and an inability or disinterest to act by local officials. The situation has been further complicated by an inefficient and disorganized international response. Excessive food aid without any insistence on guaranteeing sustainability has been cited by some authors as a perpetuating factor of this ill in Sub Saharan Africa. Certain groups are particularly vulnerable to food insecurity, including women (especially low income pregnant and lactating women), victims of conflict, the ill, migrant workers, low-income urban dwellers, the elderly, and children under five [10] Support for local and regional farming, climate prediction methods, financial aid for development and infrastructure, and a more united aid initiative would lead Sub Saharan Africa towards sustainable and reliable food sources and a more secure future. But more importantly, these solutions would lead to less dependency on foreign food aid and greater reliance on solutions from within Sub Saharan Africa. The establishment of properly functioning economic and political structures would help to lead countries to food security, as well as help to improve the overall wellbeing of the people [9]. Root causes of malnutrition in Sub - Saharan Africa

### Poverty

Childers et al estimated that some 1.4 billion people now live in absolute poverty, 40% more than 50 years ago. Nearly one of every four human beings alive today exists only on the margins of survival, too poor to obtain the food they need to work, or adequate shelter, or minimal health care, let alone education for their children [11]. Poverty is unmistakably the driving factor in the lack of resources to purchase or otherwise procure food, but the root causes of poverty are multifaceted. Poverty, combined with other socioeconomic and political problems, create the bulk of food insecurity around the globe [10]. Food distribution discrepancies happen to be a major driving factor in perpetuating lack of food in most areas of Sub Saharan Africa. Malnutrition in childhood is known to have important long-term effects on the work capacity and intellectual performance of adults. Health consequences of inadequate nutrition are enormous. It was estimated that nearly 30% of infants, children, adolescents, adults and elderly in the developing world are suffering from one or more of the multiple forms of malnutrition, 49% of the 10 million deaths among children less than 5 years old each year in the developing world are associated with malnutrition, another 51% of them associated with infections and other causes [12]. Fluctuation of prices of foods in the on a global scale is likely to affect these already disadvantaged population. They generally do not have diversified commercial food choices to provide in the world market. Their dependence once upon a time on incomes from commercial crops, almost exclusively in some areas, like Cocoa and Coffee was matched by serious suffering, malnutrition and disease when the prices of these products experienced dramatic drops in the world market [13]. Focusing on children under the age of five, who are the most affected by malnutrition in Sub Saharan Africa, a vicious cycle has been described to actually exist between poverty and malnutrition. In fact, the World Bank estimates that on average individuals suffering from malnutrition lose 10 per cent of their potential lifetime earnings. This has a much broader impact too; in the same report the World Bank found that countries can lose 2-3 per cent of their GDP because of under nutrition [13]. Malnutrition has in some instances been actually considered, and generally is considered as a poverty indicator. Malnutrition leads to sub optimal intellectual development. Knowing that children are the future of any society, an unproductive generation shall thus be prone to be poor, completing this poverty malnutrition chain [14]. Malnourished women usually have malnourished fetuses during pregnancy, delivered generally with low birth weights and consequently growing into physically and mentally stunted children. Stunted adults imply low human capital, low incomes and poverty.

The second problem is the co-existence of under- and over-nutrition in the same household, family or community. This double burden is extended to a double burden of disease. Therefore, as in many other developing countries, the over-nutrition-related diseases emerged before the battle against under-nutrition deficiency diseases has been won. This phenomenon can, at least partially, be explained by the effects of foetal malnutrition and the low quality of staple-food diets (sufficient energy but not enough micronutrients) in poor households. However, the relationship between household food insecurity and the overweight status of mothers and children are not only observed in developing countries. Several authors have found that this phenomenon is also prevalent in the developed world [15].

### Education and malnutrition

Improving the educational status of parents, especially of mothers, on nutrition, sanitation and common disease prevention strategies should logically reduce the malnutrition related mortality and morbidity. It is said that the way to the child's stomach is through the mind of the mother. Quality of food taken, choices and quantity are all at the discretion of the mother or care giver. This problem is very crucial in Sub Saharan Africa, where access to formal education for the girl child in certain communities is still a major burning challenge. The burden of malnutrition has been directly linked to poverty, quality of food intake, excessive disease and poor health status [13]. The relationship between education and poverty is too close, and virtually integrates into the virtual cycle of Ignorance, disease and poverty. Education could help reduce excessively large family sizes that are usually seen in most regions of Sub Saharan Africa. A poor community of certain cultural beliefs might not actually realize that giving birth to a fewer number of children might actually help them to match their limited resources, and also offer adequate and quality nutrition to the family.

Musgrove et al describes three important ways that ignorance and lack of education contribute to malnutrition. First people may know very little about vitamins or nutrients, and they fail to eat even the cheap and available ones. Secondly, ignorance about causes of disease and its consequences. Treatment and prevention options maybe most of the time very accessible and cheap. Poor hygienic conditions and the inability to control some intestinal parasites (Ascaris Lumbricoides and Hook worms) have serious impacts in competing for nutrients with the host, causing anemia and suppressing appetite. Huge decreases in school performance amongst children infected by these parasites have been reported. Thirdly, some people might be ignorant on how to care for their young children as they might undervalue healthy practices like breastfeeding, offering vitamins and other micronutrient rich foods to their children [16]. Improvements in women's education have contributed by far the most accounting for 43 per cent of the reduction in child malnutrition between 1970 and 1995 while improvements in per capita food availability contributed about 26 per cent [21].

### Climate change

Increasingly variable rainfall patterns are likely to affect the supply of fresh water. A lack of safe water can compromise hygiene and increase the risk of diarrheal disease, which kills 2.2 million people every year. In extreme cases, water scarcity leads to drought and famine. By the 2090s, climate change is likely to widen the area affected by drought, double the frequency of extreme droughts and increase their average duration six-fold [17]. According to the World Health Organization, Many of the major killers such as diarrheal diseases, malnutrition, malaria and dengue are highly climate-sensitive and are expected to worsen as the climate changes. The direct damage costs to health (i.e. excluding costs in health-determining sectors such as agriculture, water and sanitation), is estimated to be between US$ 2-4 billion/year by 2030 [18].

For Sub-Saharan Africa, the Comprehensive Climate Change scenario studies carried out by the International Food Policy Research Institute (IFPRI) predicts consistently higher temperatures and mixed precipitation changes for the 2050 period. Compared to historic climate scenarios, climate change will lead to changes in yield and area growth, higher food prices and therefore lower affordability of food, reduced calorie availability, and growing childhood malnutrition in Sub-Saharan Africa [19].

Climate change represents a major threat for the coming decades, particularly in Africa which has more climate sensitive economies than any other continent. Some regions in Africa have become drier during the last century (e.g. the Sahel) and it is projected that the continent will experience a stronger temperature increase trend than the global average [20]. Africa has often been identified as one of the most vulnerable regions to climate variability and change because of multiple stresses and low resilience, arising from endemic poverty, weak institutions, as well as recurrent droughts and associated complex emergencies and conflicts. Climate-related risks have significant impacts on African populations and economies and drive large allocations to emergency resources [20]. Under nutrition in turn undermines the resilience of vulnerable populations decreasing their ability to cope and adapt to the consequences of climate change and their ability grow economically.

Climate variability and change considerably influence shocks, trends and seasonality that observed and predicted in Sub-Saharan African countries, and that represent sources of stresses in the lives and livelihoods of exposed communities. Increased temperatures deplete land of its moisture more rapidly and can lead to regional water scarcity, salinization of agricultural lands, and to the destruction of crops. As temperatures increase, precipitation is becoming more variable over most of Africa. For some regions, rainfall variability and unpredictability has been substantial in the past forty to fifty years. According to Boko et al, there has been an overall annual decline in rainfall observed since the end of the 1960s over Africa with some regions experiencing greater declines than others. For instance, the Sahel and Southern Africa have become drier during the twentieth century [20].

### Government policy, political zeal and corruption

Tackling malnutrition is directly related to the achievement of Millennium Development Goal (MDG) 1 (eliminating hunger), MDG 4 (reducing child mortality) and MDG 5 (reducing maternal mortality). In fact, the achievement of many of these goals in human development hinges upon the elimination of malnutrition, as it impacts on health, productivity and educational achievement. However, most African government have either underestimated, undermined or have a lukewarm attitude with respect to investing and ensuring alleviation of malnutrition. Corruption is highest in Sub Saharan regions, with resources concentrated in the hands of a few. The fight against this ill in recent years has produced very insignificant results. Misappropriation of state funds and corruption have led to division amongst peoples, wars with massive killings, spending on expensive war equipment, further impoverishment of the population, aggravating the burden and consequences of malnutrition in this part of the world. The policies of national Governments and international institutions over past decades have neglected SSA's rural and agricultural development. Policies such as structural adjustment programs that aimed to close budget gaps, created large human development deficits, especially among the poor, and skewed allocations of national revenue and foreign aid so that agriculture and nutrition were neglected. The first attempt to address the problem of food insecurity through more than just food aid in SSA was through the “Freedom from Hunger Campaign”, initiated by the FAO and other development agencies. The campaign sought to involve developing countries in analyzing the causes of food crises and malnutrition, and to find sustainable solutions. However, nearly six decades later, that worthy intention has not been fulfilled in all parts of the world. Early attempts by African Governments to tackle the food security situation on the continent, such as the Lagos Plan of Action (1980-1985) and Regional Food Plan for Africa (1978-1990), also failed due to organizational and financial difficulties [21]. However, with the dawn of the new millennium, many African Governments have committed to increasing public spending on agriculture by signing the Maputo Declaration on Agriculture and Food Security in 2003 [22]. This however has always been a myth rather than reality, as trends continue predicting bad days with respect to hunger and malnutrition in most areas of the continent. Governments have not actually measured the burden of malnutrition, and consequently fail to consider the fight against malnutrition as a priority. Methods used at times, because of lack of adequate expertise might be outdated and ill adapted to the contexts to meet present day challenges. Genetically modified foods have been adopted in many areas of Africa with interesting results. However, many countries are still not in for it, or have not committed to testing this new method, which could be a possible solution to this ill. Ethical issues still surround the usage of genetically modified foods in most areas of the world, despite outstanding evidence of its safety [28, 29].

### Other causes

Poor distribution channels and inequalities in global food distribution: countries that have registered the highest improvements in overall malnutrition rates are not the countries that have experienced the highest growth rates, indicating that changes in malnutrition are not proportionate of economic growth. Furthermore, the countries that managed to reduce inequalities the most were not systematically the ones with the highest growth rates either, indicating that policies need to address the constraints of the most vulnerable households for growth to be both nutrition-sensitive and inclusive. Aggregate reduction in child malnutrition across countries should not conceal the fact that not all segments of the population benefit from improvement with the same magnitude. There is a need for policies that address the specific constraints of households left out of progress so that growth can be nutrition-sensitive and inclusive [23].

*"The situation described above takes place in an international context in which enough food is produced so that no child or person in the world dies from lack of food or suffers chronic malnutrition. It is inexcusable that around 1.3 billion tons of foods are annually wasted at the global level. While some 10 million children die every year from malnutrition before reaching age five, inhabitants of developed countries have the luxury of throwing away 95 to115 kg of food per capita. Many of the people living in extreme poverty spend almost 70% of their income in foodstuffs. In addition to the 2 billion people suffering from malnutrition, thousands of millions live on the verge of food insecurity and suffer the effects of increased food prices resulting from the crisis of the global capitalist system that has been imposed on us by major centers of power"* Statement by the Cuban delegation at the plenary meeting of the second committee on Agriculture Development and Food Security. New York, 6 November 2012).

### Sociocultural and religious factors

Breastfeeding practices and weaning foods are associated to malnutrition. Maternal educational level, maternal age, marital status, availability of pipe borne water and latrines have been reported to be associated to malnutrition [24]. Childhood malnutrition is accounted for by contextual effects over and above likely compositional effects, that urban-rural differentials are mainly explained by the socioeconomic status of communities and households, that childhood malnutrition occurs more frequently among children from poorer households and/or poorer communities and that living in deprived communities has an independent effect in some instances. Socioeconomic inequalities in childhood malnutrition are more pronounced in urban centers than in rural areas [25].

### Gender and malnutrition

Intra family gender inequalities in food distribution and nutritional status have been observed. For instance, in Bangladesh, 54% of malnourished children are females and have a likelihood of 1.44 times greater to be malnourished than males [26]. More often than not, the face of malnutrition is female. In households which are vulnerable to food insecurity, women are at greater risk of malnutrition than men. Malnutrition in mothers, especially those who are pregnant or breastfeeding can set up a cycle of deprivation that increases the likelihood of low birth weight, child mortality, serious disease, poor classroom performance and low work productivity [27]. According to the Food and Agricultural Organization, FAO, vulnerable women and girls are more likely to die of malnutrition than men and boys. Social and economic inequalities between men and women often stand in the way of good nutrition [27]. This condition is seen in South Asian and African communities, where boys and men are culturally selected to eat more nutritive foods such as eggs [26, 27].

## Conclusion

Despite extensive global economic growth in recent decades, including in some of the poorest countries in Africa, millions of people remain locked in a vicious cycle of hunger and poverty. Poverty means parents can't feed their families with enough nutritious food, living children malnourished. Malnutrition leads to irreversibly stunted development and shorter, less productive lives. Less productive lives mean no escape from poverty. Many African countries are even becoming more food insecure. The millennium development goal with respect to hunger eradication with respect to Africa has been a failure. Low levels of education especially in women are key perpetuators of poor nutrition practices in this Region of the World. Children under five are the most affected. Male children tend to have better health status than females in certain communities. The problem is further aggravated by adverse climatic conditions, with droughts and floods in some areas. Government policy has not been very successful most of the time, because most governments fail to consider this as a serious issue, and rescue projects from the western world are vertical and do not consider socio cultural realities of target implementation sites. In Africa, funds accorded for fighting malnutrition are often embezzled.

- Improving government policy, increasing political will and application of community adapted strategies in tackling this issue is fundamental. It should be recognized, not only as a public health issue, but as a fundamental human right especially for children to eat. Starting life disadvantaged with adverse consequences from malnutrition (ill health, mental retardation, high malnutrition related morbidity and mortality resulting especially from under-five deaths) is a neglected but serious developmental hindrance to Sub - Saharan Africa.
- The fight against corruption must cease to be lip service but actually get effective.
- The use of modern agricultural techniques to increase food production is very essential. Provision or subsidization of the ministries of Agriculture to provide fertilizers, use genetically modified foods to resist adverse weather conditions and improve yield could be possible solutions to be investigated.
- Improvement of the transport system to give access to locals to sell their local produce to raise incomes for their families is important.
- Base Line surveys to determine and understand sociocultural peculiarities of each community during implementation of particular programs are vital. Avoidance of vertical programs could be of great help.
- The ministry of environments of countries must engage in programs to protect the environment which continues to be degrading. Feasible and sustainable irrigation programs should be scaled up especially in drought affected regions.
- The solution to this problem of malnutrition in developing countries entails a multisectorial approach with well defined and achievable goals. The ministries of health, education, agricultural, environment, universities and research organizations and other non-governmental organizations or international donors must work together if any tangible outcomes are expected.

Further research involving the potential acceptability of new agricultural technologies, modern farming methods and genetically modified foods in a Sub Saharan African context should be undertaken. Understanding the socio cultural peculiarities of the milieu is fundamental. It might be difficult and unproductive implementing some health promotion programs, especially when they are very vertical and culturally inadapted. Implication of the community representatives in the programs from the early planning stages could be key determinants of program ownership, acceptability and sustainability.
